# Review of Inflammatory Bowel Disease in China

**DOI:** 10.1155/2013/296470

**Published:** 2013-11-14

**Authors:** Lingna Ye, Qian Cao, Jianfeng Cheng

**Affiliations:** ^1^Gastroenterology Department, Hangzhou Xiasha Hospital, Zhejiang 310016, China; ^2^Gastroenterology Department, Sir Run Run Shaw Hospital, Zhejiang University Medical College, Hangzhou, Zhejiang 310016, China; ^3^Division of Gastroenterology, Department of Internal Medicine, Carolinas Medical Center, Charlotte, NC 28203, USA; ^4^University of North Carolina at Chapel Hill, Charlotte Campus, USA

## Abstract

Inflammatory bowel disease mainly consisting of ulcerative colitis and Crohn's disease has been rising gradually during the last two decades in China. In this review article, we provide the latest epidemiological trends in incidence, prevalence, and mortality of IBD patients in China and summarize the risk factors and genetic susceptibility of Chinese IBD patients. We also compare these characteristics to those of IBD patients in Western countries.

## 1. Introduction

Inflammatory bowel disease (IBD) predominantly consists of two chronic, often relapsing, immunologically mediated gastrointestinal disorders: ulcerative colitis (UC) and Crohn's disease (CD). The two diseases share many clinical, pathogenetic, and epidemiologic characteristics, suggesting that underlying causes may be similar. The chronic inflammation associated with IBD is related to a sustained immune response. It is yet to be determined whether this is an appropriate response to an unknown pathogen or an inappropriate response to normal gut contents.

Crohn's disease primarily affects the terminal ileum and colon but can involve any portion of the gastrointestinal tract from mouth to anus. It is often characterized by discontinuous lesions (“skip lesions”) with inflammation that can involve the full thickness of the affected portion of bowel from mucosa to serosa.

Ulcerative colitis commonly involves the rectum, but it can extend proximally (longitudinally) to affect other portions of the colon. UC can be classified by extent of disease as proctitis, left-sided colitis, or pancolitis (when the whole colon is affected). The inflammatory lesions seen are in a continuous pattern and only affect the mucosal layers of the colon without deeper involvement.

In recent decades, the incidence of IBD in traditionally high incidence areas, such as the United States and Europe, has been relatively stable. However, the incidence of IBD has become increased in previously low incidence areas, including China. Since there is no national IBD registry, epidemiological studies are more difficult to perform in China. To our knowledge, few population-based IBD epidemiological studies are done from Mainland China. This paper will provide the latest epidemiological trends in incidence, prevalence, and mortality of IBD in China, summarize risk factors and genetic susceptibility of Chinese IBD patients, and compare these factors to those of IBD patients in Western countries.

## 2. Clinical Epidemiology

### 2.1. Epidemiology

#### 2.1.1. Incidence and Prevalence

It is estimated that UC and CD prevalence in China is up to 11.6 cases per 100,000 person-years and 1.4 cases per 100,000 person-years, respectively [1]. The prevalence may be underestimated from hospital- based data. Although the prevalence in China is lower than in the West, these figures increase rapidly. An analysis of 10218 UC cases in China suggests the prevalence for UC increase by 3.08 times from 2506 cases in 1980s to 7512 cases in 1990s [2]. Compared to 1990, the nationwide ratio of patients with UC and CD to total hospitalized patients has increased by 2.11 times [3] in 2001 and by 2.78 times [4] in 2003, respectively. In Hong Kong, the incidence of CD tripled while the incidence of UC increased slightly over the last 10 years [5]. Lok et al. [6] found that the incidence growth trend of UC was not obvious but the prevalence of UC tripled during the period of 1990 to 2006. The latest available incidence rates of UC in Hong Kong increased by 6 times over the 2 decades [7].

The above studies from China are almost all hospital-based small-scale studies, focusing on single region and inpatient cases, which can lead to underestimate the true incidence and prevalence due to some underlying selection bias. However, population-based IBD incidence and prevalence data is more reliable in Japan since it has National IBD registry. It is shown that the incidence of IBD rises tenfold [8, 9] and the prevalence is also growing in Japan. Yao et al. [10] have found that the incidence of CD in Japan quadrupled and the prevalence increased by 4.7-fold from 1986 to 1998. In Singapore the hospital-based prevalence of CD increased by 5.5-fold, during the past 15 years [11]. In Seoul, Republic of Korea, the population-based incidence of IBD also increased tenfold over the past 20 years and the ratio of UC and CD decreases from 6.8 to 2.3, indicating that the incidence of CD often overtakes UC [12].

Incidence and prevalence of UC and CD from selected geographic regions are shown in Tables 1 and 2, which suggest that the incidence of IBD increases steadily in East Asian countries, although it is lower than that in Europe and the United States [13–15]. In the Western countries, the incidence of IBD has been growing rapidly since World War II and has stabilized and even declined in some areas currently. It is anticipated that the incidence difference between Asian and Western countries will decrease and may eventually disappear.

### 2.2. Mortality

A retrospective analysis of 3100 inpatients suggested that UC mortality of mainland China was 0.6% over the 5 years follow-up period [3]. A long-term study from Hong Kong showed that the 10-year and 20-year cumulative survival rates in patients with UC in Hong Kong are 95% and 94%, respectively [7]. Another long-term study in Japan showed that the cumulative survival rates decreased year by year and decreased to 96.2% by the 10th year [19]. From the Korean studies involving 304 UC cases, the cumulative survival rates after 1, 5, 10, and 15 years are 100%, 99.4%, 97.4%, and 89.9%, respectively [20]. The 10-year cumulative survival rate (91.1%–93%) in Asian patients with UC was roughly equal to Western countries, and the mortality rate did not differ from the general population [20]. There are no large-scale long-term follow-up studies from mainland China population; the reported mortality rate from Hong Kong is consistent with Japan and Republic of Korea. 

Thus far, mortality data for CD in China remains unpublished. A Japanese cohort study involving 276 CD patients [21] reported the cumulative survival rates of 98.9% at 5 years, 98.1% at 10 years, 97.7% at 15 years, and 94.9% at 20 years after the onset of disease. This study indicated a small but persistent decline in relative survival over time, consistent with most CD survival studies in the Western world [22]. In addition, the survival studies in the Western countries suggest that age of diagnosis older than 40 years is an independent risk factor for increased mortality.

### 2.3. Colorectal Cancer

Compared to Western studies of 3–5% [22], the incidence of colorectal cancer (CRC) among Chinese UC patients had been reported to be lower. However, results varied from study to study. A retrospective analysis of 3100 hospitalized UC patients suggests that the incidence of CRC is 0.4% [3]. Another retrospective analysis of 513 hospitalized IBD patients, including 242 UC and 271 CD, shows that 4 UC patients (1.65%) developed cancer and 4 (1.65%) were confirmed with precancerous lesion, but none of the 271 CD patients developed cancer [23]. This risk is lower than results from a meta-analysis [24] of CRC risk among Western UC patients of 1.6% at 10 years, 8.3% at 20 years, and 18.4% at 30 years, and this has been thought to be due to a relatively shorter duration of disease and a lower population risk of sporadic CRC. 

### 2.4. Age and Gender Distribution

Age and gender distribution of UC and CD from different regions are shown in Table 3. In China, the mean age of onset with CD is about 10 years earlier than UC, just as in Japan, Republic of Korea, and Western countries. But the peak age of IBD onset in Mainland China is older than that in other countries. Although Asian studies reported a similar peak age of onset for both UC and CD among Japan, Republic of Korea, and Western countries, the second smaller peak is more likely to occur in Western countries [22], with the exception of Japan, which has a second smaller peak age on 60–64 years old [21]. Latest study suggests a second smaller peak age on 45–54 years old in Hong Kong [7], but not found in Mainland China.

As shown in Table 3, in China, there were more male than female patients with CD and UC [4, 5]. In studies from Western countries, there is rarely gender distribution difference. But the latest study [17] shows that in high incidence areas, UC occurs more frequently in men and CD occurs 20% to 30% more frequently in females while in low incidence areas, CD has been reported more frequently in men.

## 3. Risk Factors

### 3.1. Familial Aggregation

A family history of UC in China was previously noted to be uncommon, with a frequency ranging from 4.4% to 6.7% in Mainland China [25] and with a frequency of 2.7% in Hong Kong [6]. A recent study from Hong Kong [5] reported a familial occurrence of CD at 0%, considerably lower than the reported rates from Western series ranging from 10% to 20% [25]. In Asia, the familial occurrence of CD is ranging from 1.6% to 4.5% [22], which is similarly lower compared to that in the Caucasian population. Recent studies from China have reported a similar lower familial aggregation rate for UC, ranging from 1.5% to 5.6% [22]. Within East Asia, Japan had reported a familial occurrence of CD at 2.8% [8], and Republic of Korea had also reported a familial occurrence of CD at 2.6% and UC at 2.9% [12]. The lower familial clustering rates observed in China, Japan, and Republic of Korea are related to the low prevalence of IBD in these countries. In a recent study from Republic of Korea [12], a twofold increase (from 1.3% in 2001 to 2.7% in 2005) in the frequency of a positive family history was observed, while there was a corresponding twofold rise (from 19.81 per 100,000 in 2001 to 42.11 per 100,000 in 2005) in the prevalence of IBD. This finding suggests that the familial clustering rate of IBD may rise with time in parallel with the increase in prevalence of IBD. 

### 3.2. Cigarette Smoking

Since the first widely publicized report of an inverse association between ulcerative colitis and cigarette smoking, many studies have confirmed this unusual finding. A Chinese case-control study in China also demonstrated that smoking reduces the risk of UC [18]. However, an analysis of 10218 UC cases in China suggests that smoking does not correlate with the severity of UC, but ex smokers are more likely to encounter UC recurrence than those who continued to smoke [2]. In another study from Europe [26], smokers with ulcerative colitis who quit smoking had more active disease, more hospitalizations, and greater need for corticosteroids or azathioprine compared with those who continued to smoke. The mechanism of action for this unusual association remains unclear—potentially important effects of nicotine on rectal blood flow, colonic mucus, cytokines, and eicosanoids have been reviewed elsewhere.

It has been confirmed that smoking is an independent risk factor for CD. It was similarly shown in a Chinese population. A case-control study from China [27] has implicated cigarette smoking as a risk factor for CD. Another study from Japan [8] suggests that former smokers are also at risk, but the magnitude is less than that for current smokers. Smoking can influence the clinical course of CD. Patients with CD who smoke are more likely to have ileal than colonic or ileocolonic involvement, and smokers are more likely to have CD with penetrating or stricture disease instead of pure inflammatory CD. Continued cigarette smoking following surgical resection increases the risk of recurrent disease. 

### 3.3. Appendectomy

There is only one case-control study involving 96 cases accessed, which suggests that appendectomy has no relation to the development of UC [18]. A meta-analysis [28] of 17 case-control studies involving almost 3600 cases and over 4600 controls demonstrated a 69% risk reduction for the development of UC. Several Asian case-control studies had reported a similar protective effect of appendectomy against UC, with ORs ranging from 0.11 to 0.38 [22]. In a multicenter study from Japan [28], UC patients diagnosed after appendectomy also tended to have delayed onset, fewer relapses, and fewer colectomy compared to patients with an intact appendix.

In contrast, most studies [29] have suggested that appendectomy is a risk factor for CD development. However, children who underwent appendectomy before the age of 10 years old were less likely to develop CD. Those who developed CD following a surgery for perforated appendicitis had a more aggressive form, requiring intestinal resection at least twice as frequently as others. It remains controversial, with a recent large population-based study pointing to a diagnostic bias as the likely explanation for the association. There is only one case-control study involving 51 CD cases in China [27], which did not reveal the association of appendectomy and CD. 

### 3.4. Mycobacterial Infection

The association between mycobacterial infection and IBD remains controversial. A case-control study from China suggests that gastrointestinal and respiratory infection during childhood are risk factors for the development of CD [27]. Although several open-label studies of antibiotic regimens with antimycobacterial activity have suggested clinical improvement, the results from randomized clinical trials are less compelling [30–32]. 

### 3.5. Diet

No consensus on the association between diet and IBD has emerged because of the poor recall of diet and the possibility that diet was subconsciously altered even before formal diagnosis because of gastrointestinal symptoms. The most consistent association noted in dietary studies has been the link between increased sugar intake and IBD, especially CD. There is yet no published study on the association of high sugar intake and IBD in China. Only several small-scale case-control studies [18, 33] suggest that both high dietary fiber intake and low fat intake are protective factors for IBD. In Western countries, some epidemiologic studies have implicated that low sugar and low fat and high fiber dietary may be protective against the development of IBD. A very well spurious finding could be that patients with IBD related bowel strictures and diarrhea may restrict intake of fiber and fat.

### 3.6. Other Risk Factors

Two case-control studies conducted within Mainland China have suggested an increased risk of IBD in women who take oral contraceptive or NSAID [18, 27]. Other putative risk factors for IBD, such as perinatal and childhood factors (breastfeeding, domestic hygiene, and infection), measles infection, or vaccination remain controversial [29], and they have not been formally investigated in Chinese cohorts.

## 4. Molecular Epidemiology

Descriptive epidemiologic studies have highlighted familial aggregation as a risk factor for IBD, suggesting the genetic susceptibility plays an important role in developing IBD. Identifying these susceptibility genes and gene differences for IBD between Asian and White populations may explain some of the observed epidemiologic differences. 

Three single nucleotide polymorphisms (SNPs) of the NOD2/CARD15 gene have been first identified to be independently associated with the development of CD in Caucasians. Nucleotide-binding oligomerization domain protein 2/caspase recruitment domain protein 15 (NOD2/CARD15) mutations, may account for up to 20% of CD in the white and Jewish population [34, 35]. However, these associations were not found in studies from China (including Zhejiang, Jiangsu, and Guangdong provinces) [36], Hong Kong [37], Japan, Republic of Korea, Israeli Arabs and Turkey. A case-control study [38] involving 148 cases in Guangdong, China, has found that NOD2 P268S mutation may be associated with the age of onset, location, and complication of CD in Chinese population.

Prior studies [22] had confirmed that polymorphisms in the tumor necrosis factor (TNF) genes and HLA genes are susceptible to UC. A case-control study [39] involving 402 cases in Zhejiang province, China, has found that TNF-308A is associated with the development of UC in Chinese Han population. Another two case-control studies showed that HLA-DR gene polymorphism is associated with the phenotype of UC and also showed that HLA-DR2 allele, HLA-DRB1∗15 allele in Chinese Han population of Jilin province [40], and MICB 0106 allele in Chinese Han population of Hubei province [41] may be the susceptibility genes of UC. 

Thus far, molecular epidemiology data for IBD in China remains limited to sporadic case-control cohorts. Further nationwide studies involving multiraces are called to determine if the susceptibility genes play more important role in the development of IBD among Chinese populations.

## 5. Conclusion

In the past two decades, the number of IBD in China is growing rapidly, although it is still lower compared with the incidence data from the Western country. We hypothesize that the rising incidence of IBD in China is probably due to the combination of westernization of lifestyle, healthcare expansion/improvement, and higher recognition of the disease. As the incidence increases, the differences between China and Western population show up gradually. There is less familial aggregation, lower incidence of CRC in IBD, and less NOD2/CARD15 gene mutation in Chinese population compared to the Western population. Further studies to identify unique genetic susceptibilities and environmental influences in Chinese population are needed.

The collection of accurate epidemiologic data in China has been hampered by the lack of nationwide IBD registry. Most retrospective hospital-derived data may lead to an underestimate of the incidence rate and an overestimate of disease severity. It is difficult to know whether the current epidemiology data mainly from single province and for short period can represent the whole Chinese population because different parts of China are undergoing different rates of industrialization and westernization. The nationwide population-based epidemiologic study over a long period is called urgently.

## Figures and Tables

**Table 1:** Incidence and prevalence (per 100,000) of IBD in China.

Region	Study period	UC		CD	
		Incidence	Prevalence	Incidence	Prevalence
	1997	0.35	2.30		
Hong Kong [6]	2001	0.85	4.90		
	2006	0.40	6.99		
	1986–1989	0.8		0.3	
Hong Kong [5]	1990–1992	1.2		0.4	
	1999–2001			1.0	
	1986–1988	0.3			
Hong Kong [7]	2004–2006	1.8			
	2006	2.1	26.5		
Hong Kong [16]	2011-2012	1.66		1.31	
Macau [16]	2011-2012	1.00		0.60	
Mainland China [1, 4]	1950–2000			0.28	1.38
	1990–2003		11.6		
China (Chengdu) [16]	2011-2012	0.43		0.14	
China (Guangzhou) [16]	2011-2012	2.22		1.22	
China (Wuhan) [17]	2010-2011	1.59		0.56	
China (Xian) [16]	2011-2012	0.42		0.07	

**Table 2:** Incidence and prevalence (per 100,000) of IBD from Europe, America, and other Asian Pacific countries.

Region	Study period	UC		CD	
		Incidence	Prevalence	Incidence	Prevalence
Japan [8]	1965	0.08	5.5	0.01	0.88
	1986			0.6	2.9
Japan [10]	1991			0.9	6.3
	1998			1.2	13.5
Japan [9]	1991	1.95	18.1	0.51	5.85
Japan [18]	2005		63.6		21.2
	1986–1990	0.34		0.05	
Korea [12]	1991–1995	0.87		0.22	
	1996–2000	1.74	7.6	0.52	
	2001–2005	3.08	30.9	1.34	11.2
	1990				1.3
Singapore [11, 16]	2004				7.2
	2011-2012	0.61		0.40	
America [13, 14]	1984–1993	8.3	229.0	6.9	144.1
Europe [15]	1991–1993	10.4		5.6	
Asia [1]	2004	1.0–2.0	4.0–44.3	0.5–1.0	3.6–7.7
Australia [17]	2007-2008	11.2		17.4	
New Zealand [17]	2004	7.6		16.5	

**Table 3:** Ages and gender distribution of UC and CD.

Region	UC			CD		
	Peak age of onset (years)	Mean age of onset (years)	Ratio (male/female)	Peak age of onset (years)	Mean age of onset (years)	Ratio (male/female)
Mainland China [3, 4]	40–49	44.1	1.34 : 1	31–40	37.7	1.67 : 1
Hong Kong [6]	30–39	40.6	1.34 : 1			
Hong Kong [7]	25–34* 45–54**	40.4 37^◆^	1.07 : 1			
China [25]					37.2 Mainland 32.1 Hong Kong	2.5 : 1
Japan [10]	30–35		1.15 : 1	30–35		2.3 : 1
Korea [12]		35^◆^	0.99 : 1		21.5^◆^	2.83 : 1
Western countries [22]	30–40			20–30		

*The first peak age of onset, **the second peak age of onset, and ^◆^median age.

## References

[1] Ou Y-Q, Rakesh T, Goh KL (2006). Consensus on inflammatory bowel disease in Asia-Pacific. *Chinese Journal of Gastroenterology*.

[2] Jiang X-L, Cui H-F (2002). An analysis of 10218 ulcerative colitis cases in China. *World Journal of Gastroenterology*.

[3] Wang Y, Ouyang Q (2007). Ulcerative colitis in China: retrospective analysis of 3100 hospitalized patients. *Journal of Gastroenterology and Hepatology*.

[4] CIBDW Group (2006). Retrospective analysis of 515 cases of Crohn’s disease hospitalization in China: nationwide study from 1990 to 2003. *Journal of Gastroenterology and Hepatology*.

[5] Leong RWL, Lau JY, Sung JJY (2004). The epidemiology and phenotype of Crohn’s disease in the Chinese population. *Inflammatory Bowel Diseases*.

[6] Lok K-H, Hung H-G, Ng C-H (2008). Epidemiology and clinical characteristics of ulcerative colitis in Chinese population: experience from a single center in Hong Kong. *Journal of Gastroenterology and Hepatology*.

[7] Chow DKL, Leong RWL, Tsoi KKF (2009). Long-term follow-up of ulcerative colitis in the Chinese population. *American Journal of Gastroenterology*.

[8] Yoshida Y, Murata Y (1990). Inflammatory bowel disease in Japan: studies of epidemiology and etiopathogenesis. *Medical Clinics of North America*.

[9] Morita N, Toki S, Hirohashi T (1995). Incidence and prevalence of inflammatory bowel disease in Japan: nationwide epidemiological survey during the year 1991. *Journal of Gastroenterology*.

[10] Yao T, Matsui T, Hiwatashi N (2000). Crohn’s disease in Japan: diagnostic criteria and epidemiology. *Diseases of the Colon and Rectum*.

[11] Thia KTJ, Luman W, Ooi CJ (2006). Crohn’s disease runs a more aggressive course in young Asian patients. *Inflammatory Bowel Diseases*.

[12] Yang S-K, Yun S, Kim J-H (2008). Epidemiology of inflammatory bowel disease in the Songpa-Kangdong district, Seoul, Korea, 1986–2005: a KASID study. *Inflammatory Bowel Diseases*.

[13] Loftus EV, Silverstein MD, Sandborn WJ, Tremaine WJ, Harmsen WS, Zinsmeister AR (1998). Crohn’s disease in Olmsted County, Minnesota, 1940–1993: incidence, prevalence, and survival. *Gastroenterology*.

[14] Loftus EV, Silverstein MD, Sandborn WJ, Tremaine WJ, Harmsen WS, Zinsmeister AR (2000). Ulcerative colitis in Olmsted County, Minnesota, 1940–1993: incidence, prevalence, and survival. *Gut*.

[15] Shivananda S, Lennard-Jones J, Logan R (1996). Incidence of inflammatory bowel disease across Europe: is there a difference between north and south? Results of the European Collaborative Study on Inflammatory Bowel Disease (EC-IBD). *Gut*.

[16] Ng SC, Tang W, Ching JY (2013). Incidence and phenotype of inflammatory bowel diseae based on results from the Asia-Pacific Crohn’s and colitis epidemiology study. *Gastroenterology*.

[17] Zhao J, Ng SC, Lei Y (2013). First prospective, population-based inflammatory bowel disease incidence study in mainland of China: the emergence of “Western” disease. *Inflammatory Bowel Diseases*.

[18] CIBDW Group (2008). A case control study on the risk factors of ulcerative colitis. *Chinese Journal of Digestive Diseases*.

[19] Hiwatashi N, Yao T, Watanabe H (1995). Long-term follow-up study of ulcerative colitis in Japan. *Journal of Gastroenterology*.

[20] Sang HP, Young MK, Yang S-K (2007). Clinical features and natural history of ulcerative colitis in Korea. *Inflammatory Bowel Diseases*.

[21] Oriuchi T, Hiwatashi N, Kinouchi Y (2003). Clinical course and longterm prognosis of Japanese patients with Crohn’s disease: predictive factors, rates of operation, and mortality. *Journal of Gastroenterology*.

[22] Thia KT, Loftus EV, Sandborn WJ, Yang S-K (2008). An update on the epidemiology of inflammatory bowel disease in Asia. *American Journal of Gastroenterology*.

[23] Zhao Y-J, Yuan Y-Z (2008). A clinical study on relationship between inflammatory bowel disease and colorectal cancer. *Chinese Journal of Digestive Diseases*.

[24] Eaden JA, Abrams KR, Mayberry JF (2001). The risk of colorectal cancer in ulcerative colitis: a meta-analysis. *Gut*.

[25] Ou Y-Q, Wang Y-F, Hu R-W (2008). Epidemiology of inflammatory bowel disease in China. *Chinese Journal of Digestive Diseases *.

[26] Beaugerie L, Massot N, Carbonnel F, Cattan S, Gendre J-P, Cosnes J (2001). Impact of cessation of smoking on the course of ulcerative colitis. *American Journal of Gastroenterology*.

[27] Shi X, Zheng J, Guo Z, Chen F, Wang Z (2008). Correlated pathogenetic factors of Crohn’s disease: a case-control study. *Chinese Journal of Gastroenterology*.

[28] Koutroubakis IE, Vlachonikolis IG, Kouroumalis EA (2002). Role of appendicitis and appendectomy in the pathogenesis of ulcerative colitis: a critical review. *Inflammatory Bowel Diseases*.

[29] Loftus EV (2004). Clinical epidemiology of inflammatory bowel disease: incidence, prevalence, and environmental influences. *Gastroenterology*.

[30] Gui GPH, Thomas PRS, Tizard MLV, Lake J, Sanderson JD, Hermon-Taylor J (1997). Two-year-outcomes analysis of Crohn’s disease treated with rifabutin and macrolide antibiotics. *Journal of Antimicrobial Chemotherapy*.

[31] Borody TJ, Leis S, Warren EF, Surace R (2002). Treatment of severe Crohn’s disease using antimycobacterial triple therapy—approaching a cure?. *Digestive and Liver Disease*.

[32] Shafran I, Kugler L, El-Zaatari FAK, Naser SA, Sandoval J (2002). Open clinical trial of rifabutin and clarithromycin therapy in Crohn’s disease. *Digestive and Liver Disease*.

[33] Li Y-H, Han Y, Wu K-C (2006). Investigation on the risk factors of inflammatory bowel disease: a paired study of 72 cases. *Chinese Journal of Gastroenterology and Hepatology*.

[34] Hugot J-P, Chamaillard M, Zouali H (2001). Association of NOD2 leucine-rich repeat variants with susceptibility to Crohn’s disease. *Nature*.

[35] Ogura Y, Bonen DK, Inohara N (2001). A frameshift mutation in NOD2 associated with susceptibility to Crohn’s disease. *Nature*.

[36] Zheng J-J, Zhao N, Pang Z (2007). Assosiation of CARD15 mutation with susceptibility to CD in Chinese population. *Chinese Journal of Digestive Diseases*.

[37] Leong RWL, Armuzzi A, Ahmad T (2003). NOD2/CARD15 gene polymorphisms and Crohn’s disease in the Chinese population. *Alimentary Pharmacology and Therapeutics*.

[38] Long J, Zhi F, Zhang Y (2007). Study on mutation of NOD2/CARD15 gene in Chinese patients with Crohn’ s disease. *Chinese Journal of Gastroenterology*.

[39] Cao Q, Gao M, Zhou G (2006). Association of tumor necrosis factor polymorphisms with susceptibility to ulcerative colitis in Chinese Han population. *China Medical Abstracts*.

[40] Wang L-Y, Wang J-B, Yang J-L (2007). Study on the association between DRB1 allele polymorphism and the genetic susceptibility in patients with ulcerative colitis. *Chinese Journal of Immunology*.

[41] Li Y, Xia B, Lü M (2008). An association between MICB 0106 allele and ulcerative colitis in Chinese Han in Hubei province. *Chinese Journal of Internal Medicine*.

